# Discovery of Association Rules Patterns and Prevalence of Comorbidities in Adult Patients Hospitalized with Mental and Behavioral Disorders

**DOI:** 10.3390/healthcare9060636

**Published:** 2021-05-27

**Authors:** Sunkyung Cha, Sung-Soo Kim

**Affiliations:** 1 Department of Nursing Science, Sunmoon University, Asan 31460, Korea; skc0701@hanmail.net; 2 Department of Health Administration & Healthcare, Cheongju University, Cheongju 28503, Korea

**Keywords:** mental disorder, comorbidity, association rule, hospitalization, inpatient

## Abstract

The objectives of this study were to identify the prevalence of comorbidities of mental and behavioral disorders and to identify the association rules related to comorbidities as a way to improve patient management efficiently. We extracted comorbidities of 20,690 patients (≥19 years old) whose principal diagnosis was a mental disorder from the Korean National Hospital Discharge In-depth Injury Survey (KNHDS) between 2006 and 2016. Association rules analysis between comorbid diseases using the Apriori algorithm was used. The prevalence of comorbidities in all patients was 61.98%. The frequent comorbidities of mental and behavioral disorders were analyzed in the order of hypertensive diseases (11.06%), mood disorders (8.34%), diabetes mellitus (7.98%), and diseases of esophagus, stomach, and duodenum (7.04%). Nine major association pathways were analyzed. Significant pathways were analyzed as diabetes mellitus and hypertensive diseases (IS scale = 0.386), hypertensive diseases, and cerebrovascular diseases (IS scale = 0.240). The association pathway of diabetes mellitus and hypertensive diseases was common in subgroups of mental and behavioral disorders, excluding mood disorders and disorders of adult personality and behavior. By monitoring related diseases based on major patterns, it can predict comorbid diseases in advance, improve the efficiency of managing patients with mental and behavioral disorders, and furthermore, it can be used to establish related health policies.

## 1. Introduction

Mental health conditions are increasing worldwide, and mental health conditions and substance use disorders have increased by 13% over the past 10 years (2008–2017). Mental health conditions can affect all areas of life including performance at school or work, interpersonal relationships, and ability to participate in the community. Moreover, depression is one of the major causal factors of disabilities, and it has been reported that depression and anxiety disorders alone have adversely affected the global economy by one trillion dollars annually [1,2].

The clinical descriptions and diagnostic guidelines for ICD-10 mental and behavioral disorders define a mental disorder as “a clinically recognizable set of symptoms or behaviors associated in most cases with distress and with interference with personal functions” [3]. Mental and medical disorders existing simultaneously in a patient are called comorbidities, regardless of their causal relationship. Previous studies have reported the risk of comorbidities [1,2].

The lifetime prevalence of mental disorders among Korean adults is 25.4%, indicating that one in four citizens experiences one or more mental disorders in their lifetime [4]. The most common mental disorder was alcohol use disorder (12.2%), followed by anxiety disorder (9.3%), nicotine use disorder (6.0%), mood disorder (5.3%; major depressive disorder 5.0%), and schizophrenia spectrum disorder (0.5%) [2]. Jeon evaluated adults (≥19 years old) who used medical services at least once and reported that approximately 7.7% had mental disorders and approximately 86.8% (6.3% of the total population) had a mental disorder and a chronic disease or two mental disorders [5].

Moreover, suffering from both mental and physical disorders has complex and bidirectional characteristics. When a mental disorder is accompanied by a mental or physical problem, it has been reported that the patient suffered from exacerbated symptom burden, functional disability, reduced quality of life, higher cost, and more medical service use [6,7,8,9,10]. Prevalence of comorbidities is high among those with mood disorders including depression and anxiety disorders [5].

It is difficult to estimate the proportion of people who had a mental disorder with a comorbidity, and even if it is reported, the criteria used for symptom classification vary by country [6,11,12]. Registration of medical comorbidity is an important research domain, which is related to survival [13], and various studies have been attempted. However, prevalence varies depending on the sample size and sample collection method, and it is necessary to consider the level of classification according to the international disease classification criteria and research subjects to confirm individual comorbidity.

Studies have been conducted to identify some mental disorders (e.g., schizophrenia, depressive disorder, and bipolar disorder) and a comorbidity [6], but only limited studies examined the physical and mental comorbidities, including more inclusive mental disorders. In particular, it is necessary to analyze the pattern between the accompanying diseases and check hidden meanings beyond descriptive analyses.

As more interest has been given to big data in the field of health care, studies utilizing artificial intelligence-based machine learning, deep learning, and data mining have been actively conducted [13,14,15]. One of the most widely used association rule data mining techniques is the Apriori algorithm. It is composed of two steps: selecting rules that satisfy the minimum support and confidence and analyzing the final association rule that makes lift exceed 1 [16]. This study tried to apply the association rule and identify the pattern of comorbidities by dividing the comorbidities of discharged patients whose primary diagnosis was a mental disorder into 0 and 1 and converting them into a matrix form. It is expected that the results of this study can be used as basic data for predicting the comorbidities of mental and behavioral disorders in advance, preparing plans for patient management, and establishing health policies by monitoring related diseases centering on major comorbidity patterns. This study is limited in determining occurrence or causality of disease.

## 2. Materials and Methods

### 2.1. Design and Dataset

It is a cross-sectional study using secondary data. This study used the Korean National Hospital Discharge In-depth Injury Survey (KNHDS) as raw data. The KNHDS is nationally approved statistics, surveyed annually by the Korea Disease Control and Prevention Agency since 2005, and it collects more than 200,000 cases annually by applying the stratified two-stage cluster sampling method using hospitals with more than 100 beds and their patients as a sampling frame [17]. The survey items include demographic characteristics (e.g., sex and age), hospital visit information, diagnosis information, and operation information [18]. Diagnosis information was coded according to the 7th Korean Standard Classification of Diseases (KCD-7) based on the 10th revision of the International Statistical Classification of Diseases and Related Health Problems (ICD). A database was built using MySQL to systematically manage the data. While establishing the database, the data were classified into principal diagnosis and additional diagnosis by utilizing the metadata of the diagnosis information.

### 2.2. Study Papulation

The 2005 data, the first year of KNHDS, were excluded because the scope of collecting comorbidities was different. This study used data up to 2016, which was the latest fully organized data. The subjects of this study were patients who were discharged between 2006 and 2016, were 19 years or older at the time of discharge, and were diagnosed with a mental disorder (a principal diagnosis). The final sample size was 20,690. Organic mental disorders (F04–F09) not dementia, mental retardation (F70–F79), disorders of psychological development (F80–F89), behavioral and emotional disorders with onset usually occurring in childhood and adolescence (F90–F98), and unspecified mental disorder (F99) were excluded.

### 2.3. Principal Diagnosis and Comorbidities

For discharged patients, the principal diagnosis refers to the final diagnosis confirmed after examination to diagnose the disease. It is the most important disease that requires a doctor’s visit or treatment [19,20]. Sometimes, a new disease, not related to this, is discovered, and the additional disease that requires a lot of medical resources is selected as the principal diagnosis [19,20]. Therefore, the principal diagnosis is an important medical condition that must be registered during hospitalization [21]. This study divided the principal diagnosis into dementia (F00–F03), mental and behavioral disorders due to psychoactive substance use (F10–F19), schizophrenia, schizotypal and delusional disorders (F20–F29), mood (affective) disorders (F30–F39), neurotic, stress-related, and somatoform disorders (F40–F48), behavioral syndromes associated with physiological disturbances and physical factors (F50–F59), and disorders of adult personality and behavior (F60–F69). Comorbidity refers to an additional diagnosis diagnosed during hospitalization excluding the principal diagnosis, and some patients are not diagnosed with comorbidity [22,23]. This study classified comorbidities into 267 categories according to the intermediate classification criteria of KCD-7 based on ICD-10 and it was used for statistical analysis.

### 2.4. Statistical Analysis

R version 3.5.2 (R Foundation for Statistical Computing, Vienna, Austria), an open-source statistical tool, was used to analyze the data. Comorbidities is qualitative scale data, and the apriori algorithm was used rather than the canonical correlation analysis in consideration of the limitation of not being able to consider the causal relationship between comorbid diseases. Association rules analysis, a data mining technique, was used to analyze patterns related to comorbidities. In order to intuitively understand the association rules, mental and behavioral disorders and the sub-groups of each principal diagnosis were visualized using arulesViz. apriori of R Statistical Software was applied as an algorithm. Apriori, for searching for association rules, is the first developed representative algorithm and it is designed to focus on the frequency of occurrence [24]. Recently, it has been used in a range of fields including marketing research to promote product sales and health care (e.g., decision-making for disease diagnosis, unfair medical billing, and drug use) [25,26]. This study presented support, confidence, lift, and interest support (IS) as the results of association rules analysis, and the equation is as follows.

Support (comorbidity A → comorbidity B) refers to the probability of having diseases A and B at the same time. In other words, it is the ratio at which two comorbidities are diagnosed at the same time for the study subjects. Confidence indicates the strength of the association. Confidence (comorbidity A → comorbidity B) refers to the probability of diagnosing with comorbidity B among patients diagnosed with comorbidity A. Lift is an index used to discriminate an effective association rule. The lift (comorbidity A → comorbidity B) refers to the ratio of the probability of being diagnosed with comorbidities A and B together compared to the probability of being diagnosed with comorbidity A or comorbidity B. Therefore, the association rule is meaningful only when lift is 1 or higher [27]. The IS scale is an auxiliary index for judging the effectiveness of the association rule. It can screen out rules with low support and high lift or with low lift and high support and select only rules with high lift and support [28]. Since there were many comorbidity classification categories (267), this study first selected association rules satisfying support > 0.0 and confidence > 0.1. Among them, this study chose association rules with lift > 1, and the final items were sorted in the order of IS scale.

(1) $$\mathrm{Support}\left(\mathrm{comorbidity}\;A\to \mathrm{comorbidity}\;B\right)\;=\frac{P\left(comorbidity\;A,\;comorbidity\;B\right)}{Total\;number\;of\;patient}$$

(2) $$\mathrm{Confidence}\left(\mathrm{comorbidity}\;A\to \mathrm{comorbidity}\;B\right)\;=\frac{P\left(comorbidity\;A,\;comorbidity\;B\right)}{P\left(comorbidity\;A\right)}$$

(3) $$\mathrm{Lift}\left(\mathrm{comorbidity}\;A\to \mathrm{comorbidity}\;B\right)\;=\frac{P\left(comorbidity\;A,\;comorbidity\;B\right)}{P\left(comorbidity\;A\right)\times P\left(comorbidity\;B\right)}$$

(4) $$\mathrm{IS}\left(\mathrm{comorbidity}\;A\to \mathrm{comorbidity}\;B\right)\;=\sqrt{\mathrm{Equation}\;\left(1\right)\times \mathrm{Equation}\;\left(2\right)}$$

## 3. Results

### 3.1. Characteristics of Study Papulation

This study carried out x^2^-test to examine the distribution of comorbidities by demographic characteristics of the study subjects (Table 1). There were more female patients (57.25%) than male patients. Although male patients (62.99%) had a higher comorbidity ratio than female patients (61.44%), they were not significantly different (*p* = 0.068). Patients with comorbidity (51.19 ± 1.88) were significantly (*p* < 0.001) older than those without comorbidity (43.57 ± 16.29). The frequency of 19–44 years (44.65%) was the highest, and the results showed that a higher age group had a higher prevalence of comorbidity (*p* < 0.001). National health (79.65%) was the most common insurance type, and the prevalence of comorbidity was in the descending order of others and Medicaid 1 (*p* = 0.003). Most patients were admitted through outpatient (67.13%). However, the prevalence of comorbidity was higher in patients admitted through the emergency room (62.27%), but they were not significantly different (*p* = 0.696). Treatment results were improved (90.59%), and death (0.54%), and the results showed that 93.75% of the deceased patients had comorbidity (*p* < 0.001). The patients with comorbidity (37.72 ± 132.55) had a significantly (*p* = 0.012) longer length of stay (LOS) than those without comorbidity (32.59 ± 147.63). The mean number of comorbidities of patients with mental and behavioral disorders was 1.42, and patients with comorbidity were diagnosed with 2.30 comorbidities on average. Hospitals with 500–999 beds were most frequent (48.65%), but hospitals with 100–299 beds (66.15%) showed the highest prevalence rate (*p* < 0.001).

### 3.2. Distribution and Frequent of Comorbidities

This study performed x^2^-test to analyze the distribution of comorbidities by principal diagnosis (Table 2). The prevalence of comorbidities for mental and behavioral disorders was 61.98%. Among principal diagnoses, mood (affective) disorders (F30–F39) (31.75%) was the highest, followed by schizophrenia, schizotypal, and delusional disorders (F20–F29) (21.94%), neurotic, stress-related, and somatoform disorders (F40–F48) (20.34%), and mental and behavioral disorders due to psychoactive substance use (F10–F19) (16.71%). The prevalence of a comorbidity was highest in dementia (F00–F03) (82.59%), followed by behavioral syndromes associated with physiological disturbances and physical factors (F50–F59) (69.51%), mental and behavioral disorders due to psychoactive substance use (F10–F19) (69.45%), neurotic, stress-related, and somatoform disorders (F40–F48) (69.35%), disorders of adult personality and behavior (F60–F69) (65.60%), mood (affective) disorders (F30–F33) (59.83%), and schizophrenia, schizotypal and delusional disorders (F20–F29) (45.67%), and they were significantly different (*p* < 0.001). Patients diagnosed with dementia (F00–F03), behavioral syndromes associated with physiological disturbances and physical factors (F50–F59), mental and behavioral disorders due to psychoactive substance use (F10–F19), neurotic, stress-related, and somatoform disorders (F40–F48), and disorders of adult personality and behavior (F60–F69) showed a higher-than-mean prevalence of comorbidities, while those diagnosed with mood (affective) disorders (F30–F39) and schizophrenia, schizotypal, and delusional disorders (F20–F29) showed a lower-than-mean prevalence of comorbidities. Table 3 shows the results of frequency analysis of 267 intermediate classification codes to frequently appearing comorbidities. Table 3 presents 35 comorbidities that account for more than 1% in descending order of frequencies. The analysis results showed that hypertensive diseases (11.06%) were the most frequent, followed by mood disorders (8.34%), diabetes mellitus (7.98%), diseases of esophagus, stomach, and duodenum (7.04%), neurotic, stress-related, and somatoform disorders (6.68%), dementia (6.58%), and diseases of liver (5.31%).

### 3.3. Association Rules among Comorbidities

**Figure 1:**
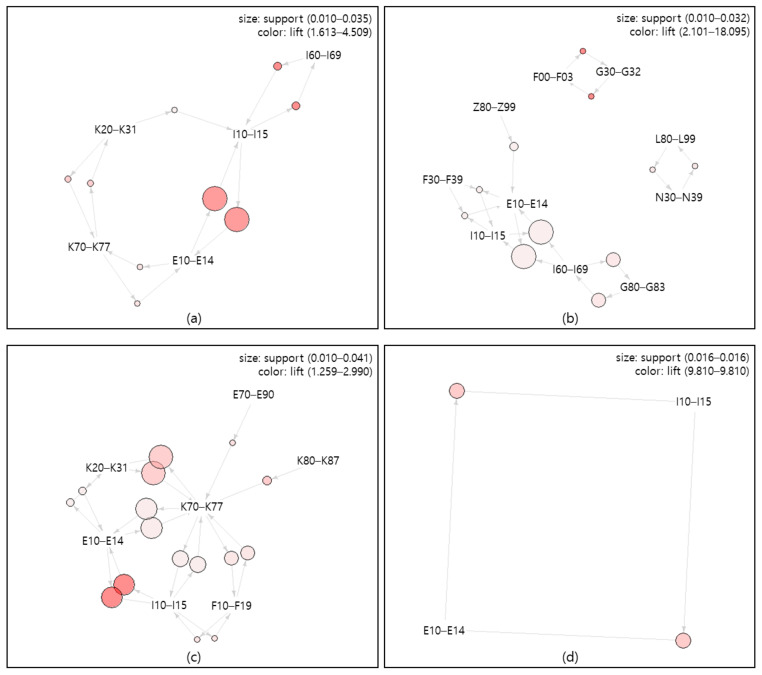

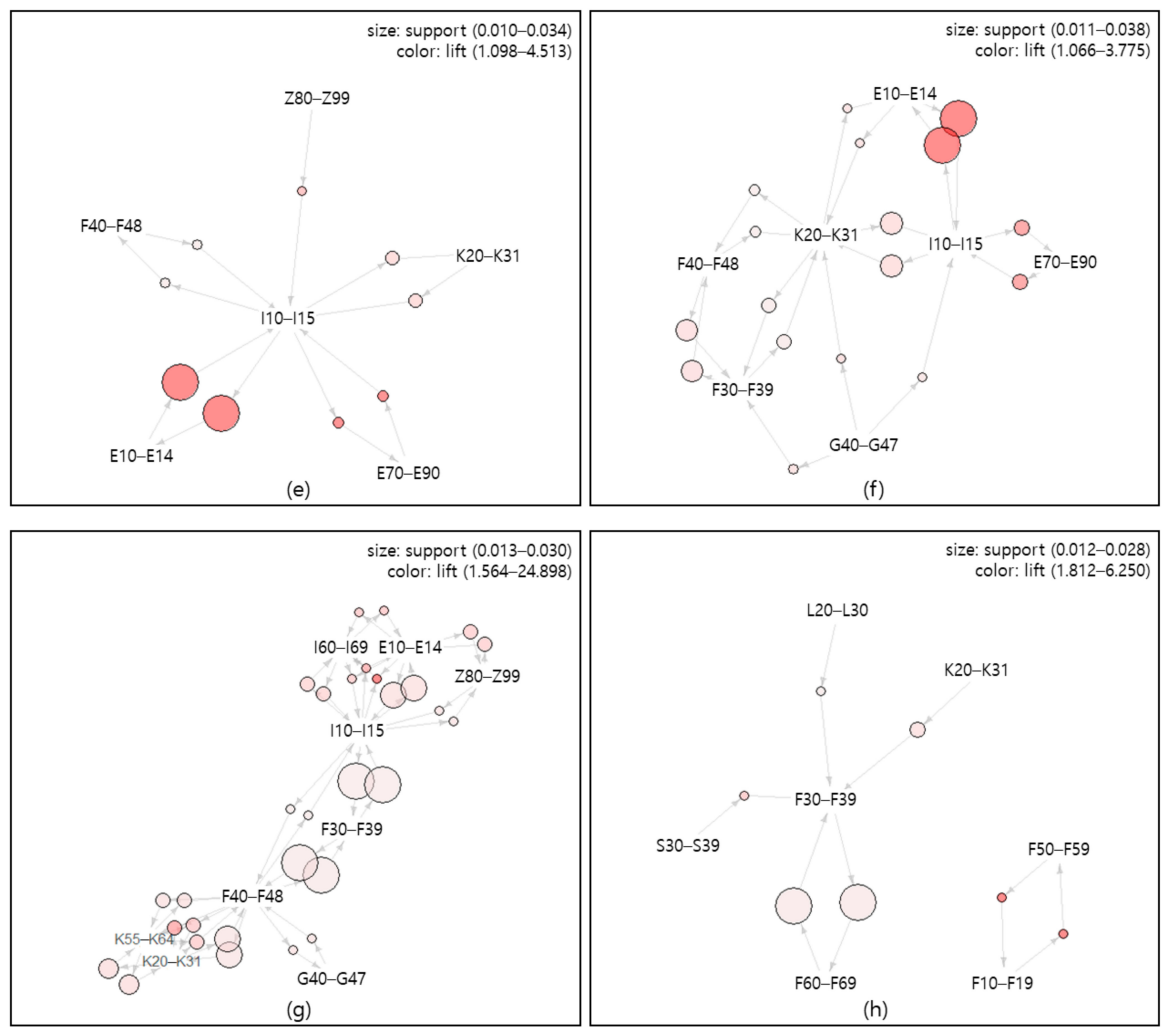
Network graph for mental and behavioral disorders and their subgroup by the principal diagnosis. (**a**) Mental disorders (F00–F69), (**b**) Dementia (F00–F03), (**c**) Mental and behavioral disorders due to psychoactive substance use (F10–F19), (**d**) Schizophrenia, schizotypal and delusional disorders (F20–F29), (**e**) Mood (affective) disorders (F30–F39), (**f**) Neurotic, stress-related and somatoform disorders (F40–F48), (**g**) Behavioral syndromes associated with physiological disturbances and physical factors (F50–F59), (**h**) Disorders of adult personality and behavior (F60–F69). ICD-10 code descriptions: E10–E14, Diabetes mellitus; E70–E90, Metabolic disorders; F00–F03, Dementia; F10–F19, Mental and behavioral disorders due to psychoactive substance use; F20–F29, Schizophrenia, schizotypal and delusional disorders; F30–F39, Mood (affective) disorders; F40–F48, Neurotic, stress-related, and somatoform disorders; F50–F59, Behavioral syndromes associated with physiological disturbances and physical factors; F60–F69, Disorders of adult personality and behavior; G30–G32, Other degenerative diseases of the nervous system; G40–G47, Episodic and paroxysmal disorders; I10–I15, Hypertensive diseases; I60–I69, Cerebrovascular diseases; K20–K31, Diseases of esophagus, stomach, and duodenum; K55–K64, Other diseases of intestines; K70–K77, Diseases of liver; L20–L30, Dermatitis and eczema; S30–S39, Injuries to the abdomen, lower back, lumbar spine, and pelvis; Z80–Z99, Persons with potential health hazards related to family and personal history and certain conditions influencing health status.

The association rules between comorbidities are visualized by using the network graph of all mental disorder patients (a) and their principal diagnosis subgroups (b~h) (Figure 1). The path of comorbidities is indicated by arrows, the size of the circle represents support, and the color of the circle represents lift. It is possible to find the association between major comorbidities by visually displaying the size and color of the circle. Dementia (F00–F03) (support 0.010–0.032, lift 2.101–18.095) formed a path with 10 comorbidities, mental and behavioral disorders due to psychoactive substance use (F10–F19) (support 0.010–0.041, lift 1.259–2.990) formed a path with 7 comorbidities, schizophrenia, schizotypal and delusional disorders (F20–F29)(support 0.016–0.016, lift 9.810–9.810) formed a path with 2 comorbidities, mood (affective) disorders (F30–F39)(support 0.010–0.034, lift 1.098–4.513) formed a path with 6 comorbidities, neurotic, stress-related and somatoform disorders (F40–F48) (support 0.011–0.038, lift 1.066–3.775) formed a path with 7 comorbidities, behavioral syndromes associated with physiological disturbances and physical factors (F50–F59) (support 0.013–0.030, lift 1.564–24.898) formed a path with 8 comorbidities, and disorders of adult personality and behavior (F60–F69) (support 0.012–0.028, lift 1.812–6.250) formed a path with 7 comorbidities.

Table 4 shows the results of analyzing the support, confidence, lift, and IS scale of the association paths between comorbidities. Although meaningful association paths are generally suggested using lift, this study presented association rules between major comorbidities by principal diagnosis subgroup in the order of IS scale considering support and lift. Among all patients with mental and behavioral disorders, the bidirectional path of diabetes mellitus (E10–E14) and hypertensive diseases (I10–I15) were the highest (IS scale = 0.386), followed by those of cerebrovascular diseases (I60–I69) and hypertensive diseases (I10–I15) (IS scale = 0.240). For dementia (F00–F03) patients, the bidirectional paths of comorbidities diabetes mellitus (E-10–E14) and hypertensive diseases (I10–I15) (IS scale = 0.472) were the strongest, followed by those of dementia (F00–F03) and other degenerative diseases of the nervous system (G30–G32) (IS scale = 0.431). The patients with mental and behavioral disorders due to psychoactive substance use (F10–F19) showed that the bidirectional paths of diabetes mellitus (E10–E14) and hypertensive diseases (I10–I15) (IS scale = 0.327) had the correlation followed by the bidirectional paths of diseases of esophagus, stomach and duodenum (K20–K31) and diseases of liver (K70–K77) (IS scale = 0.290). For schizophrenia, schizotypal, and delusional disorders (F20–F29) patients, the bidirectional path between diabetes mellitus (E10–E14) and hypertensive diseases (I10–I15) (IS scale = 0.400) was analyzed. For patients with mood (affective) disorders (F30–F39), it was meaningful in the order of the bidirectional path between persons with potential health hazards related to family and personal history and certain conditions influencing health status (Z80Z99) → hypertensive diseases (I10–I15) (Is scale = 0.171) and those between hypertensive diseases (I10–I15) and diseases of esophagus, stomach, and duodenum (K20–K31) (IS scale = 0.165). For patients with neurotic, stress-related, and somatoform disorders (F40–F48), the association was the highest in the bidirectional path of diabetes mellitus (E10–E14) and hypertensive diseases (I10–I15) (IS scale = 0.378), followed by those of metabolic disorders (E70–E90) and hypertensive diseases (I10–I15) (IS scale = 0.231). Behavioral syndromes associated with physiological disturbances and physical factors (F50–F59) patients showed a high correlation in the order of diabetes mellitus (E10–E14), hypertensive diseases (I10–I15) → cerebrovascular diseases (I60–I69) (IS scale = 0.571), and neurotic, stress-related, and somatoform disorders (F40–F48), diseases of esophagus, stomach, and duodenum (K20–K31) → other diseases of intestines (K55–K64) (IS scale = 0.524). In the case of patients with disorders of adult personality and behavior (F60–F69), it was meaningful in the order of the bidirectional paths of mental and behavioral disorders due to psychoactive substance use (F10–F19) and behavioral syndromes associated with physiological disturbances and physical factors (F50–F59) (IS scale = 0.274) and those of mood (affective) disorders (F30–F39) and disorders of adult personality and behavior (F60–F69) (IS scale = 0.234). In all subgroups of mental and behavioral disorders except for mood (affective) disorders (F30–F39) and disorders of adult personality and behavior (F60–F69), the path between diabetes mellitus (E10–E14) and hypertensive diseases (I10–I15) was found to be important.

## 4. Discussion

The prevalence and medical expenses of mental and behavioral disorders are increasing, and they are receiving a lot of attention worldwide. The comorbidities of mental and behavioral disorders are important in the management of patients with mental and behavioral disorders because they affect the duration of hospitalization, survival rate, and use of medical services [5]. Therefore, this study tried to empirically prove the association rules regarding the comorbidities of patients hospitalized due to mental and behavioral disorders using KNHDS.

The results of this study showed that the prevalence rate of comorbidities of patients with mental and behavioral disorders was 61.98%. It was lower than 67.7% of Scott et al. [29] using the nationally representative face-to-face household survey of New Zealand and 86.8% of Jeon [4] using the Health Insurance Review and Assessment Service. It is believed that the results are different because these studies included people 16 years old or older, surveyed community subjects, had a wider range of diagnostic criteria for mental and behavioral disorders, and included outpatients, unlike this study.

The prevalence of hospitalized patients due to mental and behavioral disorders was not different between male and female subjects. The results showed that older patients and patients with Medicaid 1 tended to have a higher prevalence of comorbidities. This was somewhat different from the results of Jeon [5] which indicated that the prevalence of complex mental and behavioral disorders was higher for women, older ages, and recipients of medical care. Unlike the results of previous studies, the results of this study indicated that it would be necessary to observe comorbidities of patients regardless of sex while managing health, patients, family members, and community mental health providers should raise awareness regarding comorbidities over time because the prevalence increases over time, and psychiatric health care providers need to persistently observe and follow-up for the presence of comorbidities. However, this is not a comparison of the differences between men and women for individual mental and behavioral disorders, such as mood disorders, anxiety disorders, and schizophrenia. The prevalence of all mental disorders was compared between men and women. Repeated studies are needed in the future to see if it was only targeting patients who received inpatient treatment, or whether it is a cultural characteristic of Korea in which women do not refer to symptoms as compared to men.

Considering that 93.75% of the deceased patients had comorbidities and the length of stay of patients with a comorbidity was significantly longer than that of patients without a comorbidity, managing the comorbidity of patients with mental and behavioral disorders is believed as a critical factor affecting the treatment outcomes and it is important to seek management methods to reduce the cost of treatment. Seo et al. [30] evaluated outpatients and showed that patients with chronic diseases (e.g., hypertension and diabetes) with depression stayed in the hospital longer and spent more medical expenses, and these results supported it.

The main diagnoses of hospitalization were mood (affective) disorders (F30–F39), schizophrenia, schizotypal, and delusional disorders (F20–F29), neurotic, stress-related, and somatoform disorders (F40–F48), and mental and behavioral disorders due to psychoactive substance use (F10–F19) in the order of magnitude. These mental and behavioral disorders show a high lifetime prevalence rate in South Korea [4] and are the major diagnoses in outpatient treatment [5]. Dementia had the highest prevalence of a comorbidity. It is because dementia is a senile disease, and the results suggest that it is mandatory to include comorbidity management in the dementia management system. On the other hand, mood (affective) disorders (F30–F39) and schizophrenia, schizotypal, and delusional disorders (F20–F29) showed lower prevalence rates than the mean prevalence rate of all mental and behavioral disorders. The result agreed with the results of Joo [31] that the physical comorbidity of patients with mood disorders and schizophrenia was not different from that of the healthy population. It is also believed that it is related to the results of Brink et al. [32] that the probability of detecting a comorbidity in patients with schizophrenia was less than the control group. Moreover, psychotic disorders, bipolar affective disorders, and depressive disorders included in schizophrenia, schizotypal, and delusional disorders (F20–F29) and mood (affective) disorders (F30–F39) are classified as severe mental and behavioral disorders, and it is possible that other diagnostic exploration may be insufficient because the treatment focuses on major psychological symptoms too much. Therefore, even if the prevalence of a comorbidity is low, the comorbidity of the subject should be managed with considering these the characteristics of the disease.

The results of the frequency analysis on the comorbidities of all inpatients with mental and behavioral disorders showed that hypertensive diseases, diabetes mellitus, diseases of esophagus, stomach, and duodenum, and diseases of liver among medical conditions were frequent, and mood disorders, neurotic, stress-related, and somatoform disorders, dementia, mental and behavioral disorders due to psychoactive substance use, and schizophrenia, schizotypal, and delusional disorders among mental conditions were frequent. According to a study examining the morbidity between mental and behavioral disorders in Korea, in the case of mood disorders including depression, comorbidities with other mental and behavioral disorders were most frequently observed, and comorbidities between mental and behavioral disorders were also high in anxiety disorders, sleep disorders, and schizophrenia disorders [5]. The comorbidity patterns of mood disorders and schizophrenia, mood disorders and anxiety disorders, substance-related disorders including alcohol and drug disorders and mood disorders, mood disorders and sleep disorders, and delirium/dementia and schizophrenia were high [5]. This study included both physical and mental aspects of comorbidity among adult patients hospitalized with mental and behavioral disorders.

Mental health experts who manage and provide treatment for patients with mental and behavioral disorders are needed to be fully aware of medical and mental comorbidities. Moreover, it is necessary to prepare a system that can systematically manage comorbidities. Additionally, the primary medical provider should be able to screen mental health issues and work with mental health specialists.

This study is meaningful in that it was not limited to identifying comorbidities using descriptive statistical analysis, but attempted to understand patterns between these diseases by using association rule analysis. The results of comorbidity association analysis on all mental and behavioral disorders indicated that it would be necessary to thoroughly manage the rules for major comorbidities such as hypertension, diabetes, and cerebrovascular diseases. It is needed to interconnect and manage comorbidities by introducing a hypertension, diabetes, and cerebrovascular disease registration system for patients with mental and behavioral disorders. Moreover, it is necessary to prepare plans for giving practical help such as supporting visits to doctor’s office, implementing training for enhancing self-management ability, providing visiting healthcare services, and subsidizing medical expenses. It is also needed to provide an integrated treatment and management program that manages both mental and comorbidities [30,33].

The results on the association rules of comorbidities by individual mental disorder showed that it is necessary to manage hypertensive diseases, diabetes mellitus, dementia, and the association rules of other degenerative diseases of the nervous system (G30–G32). This association rule is related to Wang et al. using the data of the Taiwan National Research Institute [34] and the Government of the United Kingdom [35], which showed that hypertension, diabetes, stroke/transient ischemic attack, coronary heart disease, and depression were found as the common comorbidities of dementia patients. It is required to consider the co-occurrence of dementia and other neurodegenerative diseases in treating mental and behavioral disorders and comorbidities comprehensively and managing dementia patients.

Mental and behavioral disorders due to psychoactive substance use (F10–F19) showed bidirectional association rules such as hypertensive diseases (I10–I15), diabetes mellitus (E10–E14), diseases of esophagus, stomach, and duodenum (K20–K31), and diseases of liver (K70–K77). It revealed that excessive alcohol intake could lead to esophageal and gastric mucosa damage, alcoholic gastritis and ulcers, duodenitis, and ulcers [36] and that chronic liver disease and cirrhosis were observed in patients with alcoholic and drug psychoses [34]. Schizophrenia, schizotypal, and delusional disorders (F20–F29) showed a bidirectional association rule between hypertensive diseases and diabetes mellitus, which was because hypertension was one of the most common comorbidities of schizophrenia patients and people with schizophrenia had an increased risk of diabetes [37].

Mood (affective) disorders (F30–F39) showed a revealed a strong association with persons with potential health hazards related to family and personal history and certain conditions influencing health status (Z80–Z99) and hypertensive diseases (I10–I15), and also same for bidirectional paths of hypertensive diseases (I10–I15) and diseases of esophagus, stomach and duodenum (K20–K31). It implied that it is important to comprehensively manage personal history, family history, various conditions influencing health conditions, hypertensive diseases (I10–I15), and diseases of esophagus, stomach, and duodenum (K20–K31) for various diseases. It is a result related to the study that the reliability of disorders of function of stomach was high in mood disorders [4].

While previous studies focused on the individual appearances of persons with potential health hazards related to family and personal history and certain conditions influencing health status (Z80–Z99), hypertensive diseases, and diseases of esophagus, stomach, and duodenum (K20–K31) in mood disorders, future studies will need to focus on their association.

Neurotic, stress-related, and somatoform disorders (F40–F48) requires managing the associations between diabetes mellitus and hypertensive diseases and between metabolic disorders and hypertensive diseases. Although the association rules of comorbidities do not imply cause or effect, systematic review and meta-analysis studies on the association between anxiety and hypertension showed that anxiety was associated with increased risk of hypertension [38] and Gigsby et al. [39] reported that 14% of adult diabetic patients had generalized anxiety disorder and 40% of them had elevated symptoms of anxiety. For behavioral syndromes associated with physiological disturbances and physical factors (F50–F59), it is needed to examine the association of diabetes with hypertensive and cerebrovascular diseases and that of other accompanying mental and behavioral disorders (F40–F48) with digestive system problems (K20–K31) and other intestinal disorders. It was supported by Choi et al. [40] who reported that the occurrence possibility of accompanying mental disorders, accompanying cardiovascular diseases, accompanying cerebrovascular diseases, and accompanying gastrointestinal disease increased with a higher severity of sleep disorders. In particular, Choi et al. [40] showed that anxiety, depression, and somatization disorder were highly associated, among accompanying mental disorders, and it supported it.

Disorders of adult personality and behavior (F60–F69), unlike other individual mental and behavioral disorders, showed meaningful association rules only with accompanying mental disorders, not physical disorders, and it was more meaningful in the order of bidirectional path between mental and behavioral disorders due to psychoactive substance use (F10–F19) and behavioral syndromes associated with physiological disturbances and physical factors (F50–F59) and between mood (affective) disorders (F30–F39) and disorders of adult personality and behavior (F60–F69). This is related to the fact that character disorder is accompanied by depressive disorder, bipolar, and related disorders, substance use disorder, eating disorder, and other types of character disorder, or patients with these disorders are often accompanied by character disorder [41]. Personality disorders in Korea are more common in outpatient treatment than in the inpatient setting, and furthermore, there are many cases without treatment, so the results of this study on inpatients are limited.

When analyzing the comorbidities of individual mental and behavioral disorders, all mental and behavioral disorders except mood (affective) disorders (F30–F39) and disorders of adult personality and behavior (F60–F69) were highly associated with hypertensive diseases (I10–I15) and diabetes mellitus (E10–E14). Even in mood (affective) disorders (F30–F39) and disorders of adult personality and behavior (F60–F69), hypertensive diseases (I10–I15) were included in the association rule. Therefore, providing various support measures by using the hypertensive diseases, diabetes mellitus, and cerebrovascular disease registration system for patients with mental and behavioral disorders mentioned above will help the management of comorbidities of individual mental and behavioral disorders. Comorbidity of psychiatric disorders did not appear to have meaningful association rules in the majority of individual psychiatric disorders, but it is thought that further research is needed.

## 5. Conclusions

This study evaluated the association rules based on the clinical evidence. The results of this study showed that major comorbidities such as hypertension, diabetes, and cerebrovascular diseases need to be managed thoroughly.

To this end, this study proposes to develop a registration and management system for the major comorbidities of mental and behavioral disorders (hypertension, diabetes, and cerebrovascular diseases) based on the association rules as a part of the mental health policy. Second, this study suggests for medical institutions to establish a management and collaboration system for the comorbidities of patients with mental and behavioral disorders and applies persistent treatment and monitoring by reflecting the registration and management system of major comorbidities on the clinical decision-making system. Third, this study suggests conducting repeated studies to analyze the association of mental and behavioral disorders and their comorbidities with expanding targets and regions by using large amounts of patient data.

The importance of this study was to evaluate the clinical validity and provide implications by empirically prove their patterns using the in-depth discharge damage survey data, which were systematically collected by the state. However, this study has limitations. First, since this study used the Korean in-depth discharge damage survey data, there are limitations in applying the results of this study to other countries. Second, due to the nature of the in-depth discharge damage survey data, the data might be biased because it excluded medical institutions with less than 100 beds. Third, despite it being the national scale estimation data, the weight was not applied in the analysis of the association rules. It is necessary to develop algorithms that allow us to overcome these shortcomings and improve the validity of the analysis. Fourth, it is needed to check association rules again even if they show a low prevalence rate of comorbidities or a low IS value depending on a mental disorder through repeated studies. Moreover, it should be managed while considering the clinical characteristics of mental and behavioral disorders. Fifth, when investigating the rules of association, this study included both comorbid mental and behavioral disorders and comorbid physical diseases. In the future, it is necessary to study by dividing into association rules related to mental and behavioral disorders or physical diseases. Lastly, it cannot be concluded that the association between comorbidities is high only by the association rules. Since this study has limitations using secondary data, we hope that a prospective study could apply various experiments, statistics, and models to find the basis for causal relationships such as randomization, control experiments, and predictive models.

## Figures and Tables

**Table 1:** Demographics and baseline characteristics of patients hospitalized with mental and behavioral disorders by comorbidity.

Variable	Comorbid Disease		Total	*x*^2^ or *t*	*p*
	With	Without			
Sex				3.342	0.068
Male	5545 (62.69)	3300 (37.31)	8845 (42.75)		
Female	7278 (61.44)	4567 (38.56)	11,845 (57.25)		
Age (year)	51.19 ± 1.88	43.57 ± 16.29	48.29 ± 17.69	−31.434	<0.001
Age group				830.972	<0.001
19–44	4836 (52.34)	4403 (47.66)	9239 (44.65)		
45–64	4607 (65.08)	2472 (34.92)	7079 (34.21)		
65–74	1856 (76.38)	574 (23.62)	2430 (11.74)		
≥75	1524 (78.48)	418 (21.52)	1942 (9.39)		
Insurance type				14.299	0.003
National health	10,116 (61.38)	6364 (38.62)	16,480 (79.65)		
Medicaid I	1972 (64.23)	1098 (35.77)	3070 (14.84)		
Medicaid II	311 (62.08)	190 (37.92)	501 (2.42)		
Others	424 (66.35)	215 (33.65)	639 (3.09)		
Admission route				0.725	0.696
Emergency	4227 (62.27)	2561 (37.73)	6788 (32.81)		
Outpatient	8589 (61.84)	5300 (38.16)	13,889 (67.13)		
Others	7 (53.85)	6 (46.15)	13 (0.06)		
Treatment outcome				89.196	<0.001
Improved	11,706 (62.46)	7037 (37.54)	18,743 (90.59)		
Not improved	973 (54.79)	803 (45.21)	1776 (8.58)		
Death	105 (93.75)	7 (6.25)	112 (0.54)		
Others	39 (66.10)	20 (33.90)	59 (0.29)		
LOS(day)	37.72 ± 132.55	32.59 ± 147.63	35.77 ± 138.50	−2.522	0.012
No. of comorbidity	2.30 ± 1.71	-	1.42 ± 1.74	N/A	N/A
Bed size				90.503	<0.001
100–299	3174 (66.15)	1624 (33.85)	4798 (23.19)		
300–499	1609 (60.58)	1047 (39.42)	2656 (12.84)		
500–999	6273 (62.32)	3793 (37.68)	10,066 (48.65)		
≥1000	1767 (55.74)	1403 (44.26)	3170 (15.32)		
Total	12,823 (61.98)	7867 (38.02)	20,690 (100)		

Abbreviations: Unit, n (%) or mean± standard deviation; LOS, length of stay; N/A, not applicable.

**Table 2:** Principal diagnosis distribution of patients hospitalized with mental and behavioral disorders with and without comorbidities.

ICD-10	Principal Diagnosis	Comorbid Disease		Total	*x* ^2^	*p*
		With	Without			
F00–F03	Dementia	1124 (82.59)	237 (17.41)	1361 (6.58)	958.193	<0.001
F10–F19	Mental and behavioral disorders due to psychoactive substance use	2401 (69.45)	1056 (30.55)	3457 (16.71)		
F20–F29	Schizophrenia, schizotypal and delusional disorders	2073 (45.67)	2466 (54.33)	4539 (21.94)		
F30–F39	Mood (affective) disorders	3930 (59.83)	2639 (40.17)	6569 (31.75)		
F40–F48	Neurotic, stress-related and somatoform disorders	2919 (69.35)	1290 (30.65)	4209 (20.34)		
F50–F59	Behavioral syndromes associated with physiological disturbances and physical factors	212 (69.51)	93 (30.49)	305 (1.47)		
F60–F69	Disorders of adult personality and behavior	164 (65.60)	86 (34.40)	250 (1.21)		
	Total	12,823 (61.98)	7867 (38.02)	20,690 (100)		

**Table 3:** Frequent comorbid diseases of patients hospitalized with mental and behavioral disorders.

ICD-10	Comorbid Disease	N	%
I10–I15	Hypertensive diseases	2289	11.06
F30–F39	Mood (affective) disorders	1725	8.34
E10–E14	Diabetes mellitus	1651	7.98
K20–K31	Diseases of esophagus, stomach and duodenum	1456	7.04
F40–F48	Neurotic, stress-related and somatoform disorders	1383	6.68
F00–F03	Dementia	1361	6.58
K70–K77	Diseases of liver	1098	5.31
G40–G47	Episodic and paroxysmal disorders	797	3.85
F10–F19	Mental and behavioral disorders due to psychoactive substance use	662	3.20
E70–E90	Metabolic disorders	590	2.85
F20–F29	Schizophrenia, schizotypal and delusional disorders	575	2.78
K55–K64	Other diseases of intestines	552	2.67
I60–I69	Cerebrovascular diseases	540	2.61
F60–F69	Disorders of adult personality and behavior	523	2.53
M40–M54	Dorsopathies	520	2.51
Z80–Z99	Persons with potential health hazards related to family and personal history and certain conditions influencing health status	503	2.43
L20–L30	Dermatitis and eczema	423	2.04
R50–R69	General symptoms and signs	398	1.92
M60–M79	Soft tissue disorders	358	1.73
M00–M25	Arthropathies	344	1.66
N30–N39	Other diseases of the urinary system	340	1.64
F50–F59	Behavioral syndromes associated with physiological disturbances and physical factors	329	1.59
E00–E07	Disorders of thyroid gland	316	1.53
J00–J06	Acute upper respiratory infections	314	1.52
I30–I52	Other forms of heart disease	271	1.31
B35–B49	Mycoses	265	1.28
K00–K14	Diseases of oral cavity, salivary glands and jaws	263	1.27
G20–G26	Extrapyramidal and movement disorders	262	1.27
J40–J47	Chronic lower respiratory diseases	254	1.23
M80–M94	Osteopathies and chondropathies	238	1.15
I20–I25	Ischemic heart diseases	236	1.14
L80–L99	Other disorders of the skin and subcutaneous tissue	227	1.10
N40–N51	Diseases of male genital organs	219	1.06
J09–J18	Influenza and pneumonia	211	1.02
J30–J39	Other diseases of upper respiratory tract	208	1.01

**Table 4:** Association rules of comorbidity of patients hospitalized for mental and behavioral disorders.

Rules	No. of Patients	Support	Confidence	Lift	IS Scale
Mental and behavioral disorders(F00–F69) with comorbid diseases (n = 20,690)					
E10–E14 → I10–I15	585	0.035	0.433	4.203	0.386
I10–I15 → E10–E14	585	0.035	0.345	4.203	0.386
I10–I15 → I60–I69	210	0.013	0.124	4.509	0.240
I60–I69 → I10–I15	210	0.013	0.465	4.509	0.240
K70–K77 → K20–K31	183	0.011	0.180	2.974	0.182
K20–K31 → K70–K77	183	0.011	0.183	2.974	0.182
K70–K77 → E10–E14	168	0.010	0.166	2.019	0.143
E10–E14 → K70–K77	168	0.010	0.124	2.019	0.143
K20–K31 → I10–I15	166	0.010	0.166	1.613	0.127
F00–F03 with comorbid diseases (n = 1361)					
E10–E14 → I10–I15	152	0.112	0.639	1.994	0.472
I10–I15 → E10–E14	152	0.112	0.349	1.994	0.472
F00–F03 → G30–G32	14	0.010	0.519	18.095	0.431
G30–G32 → F00–F03	14	0.010	0.359	18.095	0.431
I60–I69 → I10–I15	118	0.087	0.442	1.380	0.346
I10–I15 → I60–I69	118	0.087	0.271	1.380	0.346
F10–F19 with comorbid diseases (n = 3457)					
I10–I15 → E10–E14	124	0.036	0.097	2.990	0.327
E10–E14 → I10–I15	124	0.036	0.124	2.990	0.327
K20–K31 → K70–K77	143	0.041	0.089	2.032	0.290
K70–K77 → K20–K31	143	0.041	0.229	2.032	0.290
E10–E14 → K70–K77	129	0.037	0.124	1.319	0.222
F20–F29 with comorbid diseases (n = 4539)					
I10–I15 → E10–E14	74	0.016	0.035	9.810	0.400
E10–E14 → I10–I15	74	0.016	0.047	9.810	0.400
F30–F39 with comorbid diseases (n = 6569)					
Z80–Z99 → I10–I15	67	0.010	0.032	2.881	0.171
K20–K31 → I10–I15	93	0.014	0.066	1.917	0.165
I10–I15 → K20–K31	93	0.014	0.111	1.917	0.165
F40–F48 → I10–I15	76	0.012	0.095	1.098	0.113
I10–I15 → F40–F48	76	0.012	0.111	1.098	0.113
F40–F48 with comorbid diseases (n = 4209)					
E10–E14 → I10–I15	159	0.038	0.071	3.775	0.378
I10–I15 → E10–E14	159	0.038	0.140	3.775	0.378
E70–E90 → I10–I15	72	0.017	0.039	3.127	0.231
I10–I15 → E70–E90	72	0.017	0.140	3.127	0.231
K20–K31 → I10–I15	102	0.024	0.109	1.590	0.196
F50–F59 with comorbid diseases (n = 305)					
E10–E14, I10–I15 → I60–I69	4	0.013	0.023	24.898	0.571
F40–F48, K20–K31 → K55–K64	5	0.016	0.023	16.758	0.524
I10–I15, I60–I69 → E10–E14	4	0.013	0.016	15.250	0.447
F40–F48, K55–K64 → K20–K31	5	0.016	0.016	10.517	0.415
K20–K31, K55–K64 → F40–F48	5	0.016	0.020	9.776	0.400
F60–F69 with comorbid diseases (n = 250)					
F10–F19 → F50–F59	3	0.012	0.060	6.250	0.274
F50–F59 → F10–F19	3	0.012	0.032	6.250	0.274
F30–F39 → F60–F69	7	0.028	0.276	1.951	0.234
F60–F69 → F30–F39	7	0.028	0.052	1.951	0.234

Abbreviations: IS, Interest Support; ICD-10 code descriptions: E10–E14, Diabetes mellitus; E70–E90, Metabolic disorders; F00–F03, Dementia; F10–F19, Mental and behavioral disorders due to psychoactive substance use; F20–F29, Schizophrenia, schizotypal, and delusional disorders; F30–F39, Mood (affective) disorders; F40–F48, Neurotic, stress-related, and somatoform disorders; F50–F59, Behavioral syndromes associated with physiological disturbances and physical factors; F60–F69, Disorders of adult personality and behavior; G30–G32, Other degenerative diseases of the nervous system; I10–I15, Hypertensive diseases; I60–I69, Cerebrovascular diseases; K20–K31, Diseases of esophagus, stomach, and duodenum; K55–K64, Other diseases of intestines; K70–K77, Diseases of liver; Z80–Z99, Persons with potential health hazards related to family and personal history and certain conditions influencing health status.

## Data Availability

Restrictions apply to the availability of these data. Data were obtained from KCDC and are available from https://www.cdc.go.kr/contents.es?mid=a20303010502 (accessed on 8 March 2020).
